# Comparison of Serum Levels of Vitamin D and Inflammatory Markers Between Women With Gestational Diabetes Mellitus and Healthy Pregnant Control

**Published:** 2016-03

**Authors:** Fatemeh Haidari, Mohammad-Taha Jalali, Nahid Shahbazian, Mohammad-Hossein Haghighizadeh, Elham Azadegan

**Affiliations:** 1 Nutrition and Metabolic Diseases Research Center, Department of Nutritional Sciences, School of Paramedical Sciences, Ahvaz Jundishapur University of Medical Sciences, Ahvaz, Iran; 2 Laboratory Sciences Department, School of Paramedical Sciences, Ahvaz Jundishapur University of Medical Sciences, Ahvaz, Iran; 3 Department of Obstetrics and Gynecology, Imam Khomeini Hospital AND Departmet of Endocrinology, Diabetes Research Center, School of Medicine, Ahvaz Jundishapur University of Medical Sciences, Ahvaz, Iran; 4 Department of Statistics, School of Public Health, Ahvaz Jundishapur University of Medical Sciences, Ahvaz, Iran; 5 Department of Nutritional Sciences, School of Paramedical Sciences, Ahvaz Jundishapur University of Medical Sciences, Ahvaz, Iran

**Keywords:** Gestational Diabetes, TNF-α, HS-CRP, HOMA-IR, Vitamin D

## Abstract

**Objective:** Vitamin D appears to be involved in regulation of glycemic and inflammatory responses in gestational diabetes. The purpose of this study was to compare the serum levels of 25-hydroxyvitamin D (25(OH)D), inflammatory biomarkers and glycemic profile between gestational diabetes mellitus (GDM) and normal glucose tolerance (NGT) pregnant women.

**Materials and methods:** In this cross-sectional study, fasting serum levels of 25(OH)D, insulin, glucose, HOMA-IR, hs-CRP and TNF-α were measured in 45 GDM and 45 NGT women at week 20-30 gestation whom referred to Reference Medical Laboratory of Ahvaz, Iran in 1394.

**Results:** Serum 25(OH)D levels were significantly lower (p = 0.003 ) in the GDM group compared to the NGT group which remained even after controlling for confounders. Insulin and TNF-α levels were not statistically different between groups (p > 0.05). However, in unadjusted model, HOMA-IR and hs-CRP were significantly different between groups that disappeared in adjusted model. In the GDM group, there was a negative significant correlation between 25 (OH) D and fasting blood sugar (p = 0.009) and pre pregnancy BMI (p < 0.001). Levels of 25(OH)D were also negatively correlated with pre pregnancy BMI (p < 0.001) and hs-CRP levels (p = 0.003) in the NGT group.

**Conclusion:** The lower level of vitamin D may be responsible for impairments of some glycemic and inflammatory markers in pregnant women. This is more important in overweight pregnant women. However, further studies with larger sample size are recommended in this regards.

## Introduction

Gestational Diabetes Mellitus (GDM) is a disorder characterized by variable severity of glucose intolerance with onset or first recognition in pregnancy (1). GDM is a prevalent complication in pregnancy which becomes apparent in approximately 7% of all pregnancies in the world (2) and 4.9% in Iran (3). Pregnancy is a physiologic but diabetogenic condition since the steroid hormones rise and insulin resistance occurs in peripheral tissues (4). Additionally inflammatory cytokines are secreted from both adipose tissue and placenta that can contribute to insulin resistance and pathogens of GDM (5). GDM has long term and short term detrimental consequences on mother and offspring’s health (6). Some adverse effects on mother’s health include pre-eclampsia, urinary tract infection, higher risk of hypertension and development of type 2 diabetes mellitus (T2DM) after pregnancy (1, 7-11) and higher rates of GDM in future pregnancies (9). Macrosomia, birth trauma, neonatal jaundice, respiratory distress syndrome, hypoglycemia (1, 8, 9), and future risk of obesity, T2DM and impaired glucose tolerance are some adverse effects of GDM on infant’s health (12). In addition to some known risk factors such as ethnic group, prior GDM, history of miscarriage, history of T2DM in first degree relatives, obesity and hypertension which contribute to development of GDM (7, 8), recent studies have shown that vitamin D deficiency may be a modifiable contributor in the etiology of GDM (7, 12, 13). Vitamin D deficiency is prevalent in pregnancy (11). New studies expressed that vitamin D plays a key role in glucose homeostasis and insulin resistance (11, 12, 14-16). Furthermore, the anti-inflammatory effect of vitamin D in diabetes has been reported in some studies (15, 17). Probably, the active form of vitamin D decreases expression of pro-inflammatory cytokines such as IL-6, IL-1 and TNF-α involved in insulin resistant (18). Moreover, there is vitamin D nuclear receptor in some particular tissue in pregnancy such as placenta and deciduas (19) which draws more attention to its possible role in GDM. Based on previous studies, vitamin D deficiency appears to be involved in the pathogenesis of GDM (7, 12, 13) via inducing insulin resistant (11, 12, 14, 15) and inflammation (15, 17, 18). At present, there is limited evidence to investigate the relationship between vitamin D status and inflammation in GDM, and the results are very inconsistent. Therefore, the objective of this study was to compare the serum levels of vitamin D and inflammatory markers between women with gestational diabetes mellitus and healthy controls.

## Materials and methods

This cross sectional study was conducted on two groups of pregnant women (GDM and healthy control) in Ahvaz, Iran. On the basis of the similar study (16), considering r = -0.33 and power = %90 and confidence coefficient = %95 and using NCSS software, a sample size of 45 was determined for each group (GDM and healthy control pregnant women). The study participants were selected from pregnant women referred to the Reference Medical Laboratory of Ahvaz, Iran, in 1394, for GDM screening. From a total of 430 pregnant women who met all inclusion and exclusion criteria, 45 cases of GDM were diagnosed. We established a random sample of 45 women who were not diagnosed with GDM as control. Oral Glucose Tolerance Test (OGTT) with 75 gram dextrose monohydrate was performed in the morning after fasting for at least eight hours overnight for all participants. Diagnosis of GDM was confirmed if one of the glucose levels exceeded the IADPSG criteria: fasting ≥ 92 mg/dl, 1-hour ≥ 180 mg/dl, 2-hour ≥ 153mg/dl (2). We selected the case group before they were informed about the results, so no one used insulin or medications or diet to treat diabetes. The control subjects were matched to the cases for the exclusion and inclusion criteria. Inclusion criteria were as follows: aged 18-35 years, gestational age 20-30 weeks, pre pregnancy body mass index (BMI) 18.5-29.9, singleton pregnancy, non-history of use of corticosteroid, calcitonin, cytotoxic and immunosuppressive drugs, anticonvulsants, vitamin D and calcium in last three months (which affect on vitamin D metabolism). Exclusion criteria included pre pregnancy BMI ≥ 30, T1DM or T2DM, acute or chronic inflammatory diseases such as inflammatory bowel disease, metabolic bone disease, history of miscarriage and major congenital fetal anomaly in previous pregnancies, kidney, liver, thyroid and parathyroid disease, pre-eclampsia and smoking. The study was approved by the ethic committee of Ahvaz University of Medical Sciences (Act No.AJUMS.REC.1392.262). A written informed consent was obtained from all participants after giving full information about the study design and objectives.

Demographic and anthropometric information were recorded for each person. Age, gestational age, education, history of T2DM in first degree relatives, prior history of GDM and pre-pregnancy weights were noted through face to face interview. A semi-quantitative Food Frequency Questionnaire (FFQ) was used for determining vitamin D intake (20). A valid sunlight exposure questionnaire was also completed for all participants. According to previous studies, the duration of sun exposure was categorized as follows: less than 10 minutes, 10 minutes to 1 hour, 1-2 hour and more than 2 hours (21). Weight was measured to the nearest 0.1 kg with light clothes and without shoes by a digital scale (Seca). Height was measured to the nearest 0.1 cm by a tape measure attached to the scale (Seca). BMI was calculated using the following formula: BMI = Weight (kg) / [Height (m) x Height (m)]. The amount of weight gain during pregnancy was computed by subtracting pre-pregnancy weight and current weight. 5ml blood samples were obtained after an overnight fast. Blood samples were centrifuged and serum were separated and stored at -70˚ centigrade until analysis. Serum high-sensitivity C-reactive protein (hs-CRP) was assayed using ELISA kit (DBC, Canada). Serum TNF-α was quantified using ELISA kit (Boster, USA). Serum 25-hydroxyvitamin D (25(OH)D) concentrations was also determined using commercial ELISA kit (bioactivadiagnostica, Germany). Vitamin D status was categorized as sever deficiency (< 10 ng/ml), deficiency (10-20 ng/ml) and insufficiency (20-30 ng/ml) (22). In this study, serum glucose was measured by glucose oxidase kit (Bionik, Iran). Serum insulin was determined using ELISA kit (Monobind, USA). Homeostasis model assessment of insulin resistance (HOMA-IR) index was used to assess Insulin resistance and calculated using the following equation:

HOMA-IR = fasting plasma glucose (mmol/L) × serum insulin (mcIU/mL)/22.5. HOMA-IR values lower than 3 are normal. Equal to or more than 3 show sever insulin resistance   (23).

All data were represented as mean ± standard deviation (SD) and proportions. At first, the normal distribution of variables was tested by kolmogorov-smirnov test. To compare parametric and nonparametric data, independent sample T-test and Mann-Whitney test were applied respectively. ANCOVA was used to adjust for the effect of confounding variables including age, family history of T2DM and previous GDM history, pre pregnancy BMI, weight at 20-30 weeks of gestation and number of pregnancy. Chi square test and Fisher^’^s exact test were also used for categorical variables. The relationship between serum 25(OH)D levels and other variables was assessed using Pearson correlation. All statistical analyses were administered using SPSS version 16 and p < 0.05 was considered statistically significant.

## Results

Anthropometric and demographic data of the GDM and control groups are shown in Table 1. No significant differences were observed in the age, gestational age, total weight gain during pregnancy and weekly weight gain (p > 0.05).

**Table 1:** Anthropometric and demographic data of the GDM and NTD group

Variables	GDM group (n = 45)	NGT group (n = 45)	p value
**Age (year)**	**29.33 ± 4.31**	**27.51 ± 4.87**	**0.064**
**Gestational age (week)**	**26.4 ± 2.47**	**25.93 ± 3.34**	**0.453**
**Pre-pregnancy BMI**	**25.74 ± 3.05**	**24.31 ± 3.02**	**0.028**
**Current weight (kg)**	**75.08 ± 11.3**	**70.48 ± 9.67**	**0.041**
**Weight gain during pregnancy (kg)**	**5.29 ± 5.70**	**5.98 ± 4.3**	**0.518**
**Weight gain per week (kg)**	**0.19 ± 0.21**	**0.22 ± 0.16**	**0.446**
**Number of pregnancy**	**2.33 ± 1.27**	**1.49 ± 0.62**	**0.000**
**T2DM history in first degree relatives (%)**	**44.4**	**24.4**	**0.046**
**GDM history** **in previous pregnancies (%)**	**17.8**	**2.2**	**0.030**
**Duration of sunlight exposure**			**0.917**
** Less than 10 minutes** **(%)**	**20.0**	**26.6**	
** 10-60 minutes (%)**	**75.6**	**62.2**	
** 60-120 minutes (%)**	**4.4**	**11.1**	
**Dietary intake of vitamin D (mcg/d)**	**4.38 ± 2.36**	**4.90 ± 2.72**	**0.331**

Quantitative and qualitative data were expressed as mean ± SD and proportions, respectively. Independent sample T-test and Fisher’s exact test were used for quantitative and qualitative values, respectively. GDM: gestational diabetes mellitus; NGT: normal glucose tolerance; T2DM: type 2 diabetes mellitus

However, the number of pregnancy, pre pregnancy BMI and current weight were significantly higher in the GDM group compared to the NGT control. Family history of T2DM and previous GDM history were also significantly higher in the GDM group compared to the control (p = 0.046 and p = 0.030, respectively). The duration of sunlight exposure and dietary intake of vitamin D were not different between groups (p = 0.917 and p = 0.331, respectively).

The comparison of biochemical characteristics of the GDM and NGT groups are shown in Table 2. According to the results, the serum levels of 25(OH)D were significantly lower in the GDM group compared to the NGT group. This result remained significant even after controlling for confounders. Fasting blood sugar and HOMA-IR were also significantly higher in the GDM group (p< 0.001 and p = 0.022, respectively). However, it was not significant for HOMA-IR in the adjusted model (p = 0.132). The serum levels of hs-CRP and TNF-α were higher in the GDM group compared to the control (p = 0.011 and p = 0.059, respectively). However, these differences were not significant after adjusting for confounders.

The associations of serum 25(OH)D levels with anthropometric and biochemical indices are shown in Table 3. In the GDM group, the serum levels of 25(OH)D were negatively correlated with pre pregnancy BMI, fasting blood sugar, insulin, HOMA-IR and hs-CRP. In the NGT group, serum 25(OH)D levels were also negatively correlated with pre pregnancy BMI and hs-CRP levels. However, the relationship between 25(OH)D levels and other biochemical markers were not significant in this group.

In the present study, the participants were categorized using the vitamin D nutritional status into three groups including severe vitamin D deficiency (< 10 ng/ml), deficiency (10-20 ng/ml) and insufficiency (20-30 ng/ml), respectively (Table 4). The results showed a 64.4% prevalence of deficiency and 20.0% sever deficiency in the GDM group.

## Discussion

The purpose of this study was to compare the serum levels of vitamin D and some inflammatory biomarkers between GDM and NGT pregnant women. We found a significant difference in serum 25(OH)D between groups which was in agreement with other studies. Maghbooli et al.   (23) in a cross sectional study showed that serum level of 25(OH)D were significantly lower in GDM compare to NGT women and the prevalence of GDM was higher in those with sever vitamin D deficiency.

**Table 2:** Comparison of biochemical characteristics between GDM and NTD groups

Variables	GDM group (n = 45)	NGT group (n = 45)	P1	P2
**Fasting Blood glucose (mg/dl)**	**93.48 ± 10.55**	**78.77 ± 7.09**	**0.000**	**0.000**
**Fasting Blood Insulin (mcIU/ml)**	**8.40 ± 3.54**	**8.12 ± 2.88**	**0.677**	**0.917**
**HOMA-IR**	**1.95 ± 0.94**	**1.56 ± 0.56**	**0.022**	**0.132**
**TNF-α (pg/ml)**	**39.67 ± 25.15**	**29.54 ± 23.97**	**0.059**	**0.367**
**hs-CRP (mcg/ml)**	**8.02 ± 2.89**	**6.26 ± 3.48**	**0.011**	**0.171**
**25(OH)D (ng/ml)**	**13.46 ** **± 5.18**	**16.97 ± 5.56**	**0.003**	**0.034**

All data were expressed as mean ± SD. P1 were resulted from independent sample t-test and P2 were resulted from ANCOVA in the adjusted models (adjusted for age, family history of T2DM and previous GDM history, pre pregnancy BMI, weight at 20-30 weeks of gestation and number of pregnancy). GDM: gestational diabetes mellitus; NGT: normal glucose tolerance; T2DM: type 2 diabetes mellitus; HOMA-IR: homeostasis model assessment of insulin resistance; TNF-α: tumor necrosis factor–alpha; hs-CRP: high sensitive- C reactive protein.

**Table 3:** Association of 25(OH)D level with anthropometric indices and biochemical markers

Variables	GDM group (n = 45)		NGT group (n = 45)	
	r	p	r	p
**Pre pregnancy BMI**	**-0.737**	**0.000**	**-0.669**	**0.000**
**Fasting blood sugar**	**-0.395**	**0.009**	**-0.020**	**0.897**
**Fasting blood Insulin**	**-0.291**	**0.058**	**-0.065**	**0.677**
**HOMA-IR**	**-0.353**	**0.020**	**-0.08**	**0.609**
**TNF-α**	**-0.330**	**0.350**	**-0.117**	**0.466**
**hs-CRP**	**-0.337**	**0.027**	**-0.444**	**0.003**

P values were resulted from Pearson correlation. GDM: gestational diabetes mellitus; NGT: normal glucose tolerance; T2DM: type 2 diabetes mellitus; HOMA-IR: homeostasis model assessment of insulin resistance; TNF-α: tumor necrosis factor–alpha; hs-CRP: high sensitive- C reactive protein.

**Table 4:** Vitamin D nutritional status in the GDM and NGT groups

Vitamin D status	GDM group (%)	NGT group (%)	p value
**Severe Deficiency (< 10ng/ml)**	**20.0**	**8.9**	**0.301**
**Deficiency (10-20 ng/ml)**	**64.4**	**71.1**	
**Insufficiency (20-30 ng/ml)**	**11.1**	**15.6**	

All data were expressed as percent. P value was resulted from Fisher’s exact test.

Wang et al., (11) indicated a significant difference in serum 25(OH)D levels between GDM and NGT pregnant women even after controlling for age and pre pregnancy BMI and reported a 96.25% prevalence of vitamin D insufficiency and 52.75% deficiency in GDM group. According to previous studies, vitamin D deficiency is common in pregnant women (11). In the present study, vitamin D deficiency was also observed in spite of plenty of sunshine in Ahvaz, which is almost identical to Saudi Arabia, Kuwait (24) and Egypt(25) where vitamin D deficiency is prevalent. It seems that skin hyperpigmentation, avoiding exposure to sunlight, usage of clothes and covering most parts of skin (24) and air pollution (26) may result in vitamin D deficiency in these area. Low dietary intakes of vitamin D and loss of fortified foods (26) may be another causative factor in Ahvaz, Iran.

The insulin resistance during normal pregnancy is very common (27, 28). By development of pregnancy, postprandial serum glucose levels increase due to decreased insulin response and sensitivity (29, 30), and insulin-mediated glucose disposal may decline by 50% (28, 30). Also insulin secretion increase by 200-250% which is needed to maintain normal glycaemia (30). As expected, in the present study fasting blood sugar and HOMA-IR were higher in the GDM group compared to the NGT group. Several studies reported similar results in GDM women (10, 11). However, in this study, there was not a significant difference in the serum level of insulin between groups. It may be due to the impairment of insulin action, rather than insulin secretion, in GDM patients similar to T2DM patients. It can consequently resulted from declining of insulin receptors and impairing of glucose transport into cell via GLUT4 (glucose transporter 4) (31). 

The present study showed that after controlling for pre pregnancy BMI and weight at 20-30 gestational week, there was not a significant difference in the levels of inflammatory markers between groups. Similarly, McManus et al.  (32), reported no difference in TNF-α and hs-CRP between GDM and NGT women at week 31 gestation and declared that it might be due to the matched weight control. The results of other studies were very inconsistent, in this regard. Rota et al. (33), investigated the relationship between low grade systematic inflammation and GDM in non obese pregnant women at mid-gestation. They found that serum hs-CRP levels were significantly higher in GDM women despite of no difference in the pre pregnancy BMI. Also they found that the main factors which affect hs-CRP levels were glucose intolerance and weight gain during pregnancy. Moreover, it is important to note that during pregnancy, inflammatory mediators are secreted both from adipose tissue and placenta (34, 35). It may play a role in the development of low-grade inflammation during third trimester of pregnancy (34). It is confirmed that TNF-α by inhibiting insulin receptor tyrosine kinase activity in adipocyte (31) and hs-CRP by increasing free fatty acid synthesis in liver and interfering with LPL activity, take part in insulin resistant (33). Therefore, no difference in insulin resistance in the present study may be partially due to the no difference in inflammatory biomarkers.

In the present study, we found a significant negative correlation between 25(OH)D with pre pregnancy BMI in both GDM and NGT groups. Park et al., (29) and Daniel et al., (36) also reported a negative relationship between BMI and serum 25(OH)D levels. These investigators showed that when serum 25(OH)D levels were improved from deficiency to sufficiency status, the mean of BMI was declined. A recent investigation confirmed that in overweight and obese people, despite of the higher intake of energy, the intake of micronutrients, like vitamin D, is lower than normal weight subjects (37). In our study, the mean intake of vitamin D in both groups was lower than Dietary Reference Intakes (DRI). Furthermore, vitamin D is soluble in fat, therefore, the higher deposition of vitamin D in adipose tissue may also be responsible for the lower its serum levels in obese patients (29). It is important to note that the duration of sunlight exposure in this study was too insufficient, and it may be another causative factor for vitamin D deficiency.

In this study, glycemic indices (fasting blood sugar, insulin and HOMA-IR) were also negatively correlated with serum levels of 25(OH)D in the GDM group. Similar results were also reported in Maghbooli et al., study   (23), which conducted on 741 Iranian pregnant women at 24-28 gestational week. According to the previous studies, it seems that vitamin D activity is needed to maintain calcium membrane flux and appropriate insulin mediated intra cellular process (38, 39). Moreover, the presence of vitamin D receptor in pancreatic β-cell, muscle and adipose tissue (38), and vitamin D role in the regulation of insulin receptor gene transcription and in the stimulation of insulin mediated glucose transport via GLUT4 were demonstrated in other studies (11, 38, 39). 

In the present study, a significant negative relation between 25(OH)D and hs-CRP levels was observed in both groups. The results of other studies are not consistent in this debate. Mc Manus et al.,  (32) investigated the relation between 25(OH)D and adipocytokine in GDM and NGT pregnant women and reported no significant relation between these markers. However, Ngo et al., (40) observed a negative significant relationship between 25(OH)D and hs-CRP in healthy participants. The probable mechanism, which vitamin D interferes with inflammatory process, may be the suppression of nuclear factor kappa-β (NFK-β) by vitamin D that result in inhibition of endogenous CRP synthesis (40, 41).

Since the main objective of this study was focused on the comparison between GDM women and healthy controls, the present study was designed as a cross-sectional study on two groups of pregnant women. The cross-sectional design that prohibited the assessment of temporal and thus potential causal relations and the relatively small sample size could explain the lack of statistical significance for correlation results. Therefore, subsequent studies with larger sample size are needed in this regard. However, the important strength of this study was the assessment of both duration of sunlight exposure and dietary intake of vitamin D, which can affect on the serum 25(OH)D level. 

In conclusion, vitamin D deficiency was prevalent in both GDM and healthy control pregnant women. The lower serum levels of 25(OH)D and the higher levels of hs-CRP and TNF- α were shown in the GDM group compared to the control. The serum levels of 25(OH)D were also negatively correlated with pre pregnancy BMI, fasting blood sugar, insulin, HOMA-IR and hs-CRP in GDM pregnant women. It seems that vitamin D plays a role in the regulation of glycemic and inflammatory responds in GDM. However, future studies are needed to investigate the effect of vitamin D supplementation on inflammatory biomarkers and glycemia in diabetic pregnant and non-pregnant women.

## Conclusion

In conclusion, vitamin D deficiency was prevalent in both GDM and healthy control pregnant women. The lower serum levels of 25(OH)D and the higher levels of hs-CRP and TNF- α were shown in the GDM group compared to the control. The serum levels of 25(OH)D were also negatively correlated with pre pregnancy BMI, fasting blood sugar, insulin, HOMA-IR and hs-CRP in GDM pregnant women. It seems that vitamin D plays a role in the regulation of glycemic and inflammatory responds in GDM. However, future studies are needed to investigate the effect of vitamin D supplementation on inflammatory biomarkers and glycemia in diabetic pregnant and non-pregnant women.
